# Editorial: The impact of learning to read on visual processing

**DOI:** 10.3389/fpsyg.2015.00985

**Published:** 2015-07-14

**Authors:** Tânia Fernandes, Régine Kolinsky

**Affiliations:** ^1^Faculdade de Psicologia, Universidade de LisboaLisboa, Portugal; ^2^Fonds de la Recherche Scientifique - FNRSBrussels, Belgium; ^3^Unité de Recherche en Neurosciences Cognitives, Center for Research in Cognition and Neurosciences, Université Libre de BruxellesBrussels, Belgium

**Keywords:** the impact of learning to read on visual processing, literacy acquisition, reading development, developmental dyslexia, visual processing, visual object recognition

In 1892, Déjerine published the first report of pure alexia (Déjerine, 1892). *Monsieur C*. became unable to read in the absence of any other cognitive disorder (even writing was preserved) after a lesion of the inferior occipitotemporal cortex, a neural region dedicated to visual recognition. Although reading is an intense visual ability, the relation between reading and visual processing has often been sell short. It was only ~100 years after the report of Monsieur C. that part of this occipitotemporal region was coined *visual word-form area*, VWFA (Warrington and Shallice, 1980; see also Cohen et al., 2000; Polk and Farah, 2002). Since then an emergent bulk of research has demonstrated that learning to read, not only leads to the emergence of a specialized neurocognitive circuitry, but also impacts on the evolutionary older and pre-existing neurocognitive system of visual (non-linguistic) object recognition. Many questions regarding the exact nature, locus, and consequences of this impact are in debate or still unanswered. This Research Topic was aimed at setting a landmark forum on which researchers present and discuss recent work, their proposals, and open novel questions. We have compiled nine excellent articles on the relation between visual processing and literacy acquisition, reading development, and developmental dyslexia. This research topic is organized into three parts.

In the first part, opening this research topic, in an opinion article, Zhou et al. (2014) consider the relation between visual skills and learning to read, and the moderator role of the visual complexity of the written script in this equation (e.g., Chinese makes stronger demands of visual skills due to its complexity than alphabetic scripts). Qian and Bi (2014) argue that the visual complexity of the script modulates the expression of visual processing deficits (namely, in magnocellular processing) in developmental dyslexia. They examined the association between motion processing (in a coherent motion task, underpinned by V5/MT functioning) and reading (in a visual lexical decision task) in Chinese dyslexic children and chronological-age controls.

Second, regarding the emergence of a neurocognitive system specialized in letter processing, in a hypothesis and theory article, Lachmann and van Leeuwen (2014) propose the *functional coordination* approach. According to this hypothesis learning to read captures the analytic strategy of visual processing, which was already available before literacy took place, but then becomes the preference mode in letter processing. In their research article, Lachmann et al. (2014) used the Navon test to examine whether, when the hierarchical stimulus (a global figure composed of local figures) is presented at fixation with dimensions close to those in written text, letters compared to non-letters are processed using an analytic strategy instead of the usual holistic strategy adopted on hierarchical stimuli.

In the last part of this Research Topic, the impact of literacy on non-linguistic visual processing is considered. Indeed, according to the *neuronal recycling hypothesis* (Dehaene, 2009) the ventral occipitotemporal regions, originally devoted to object recognition, are partially recycled to accommodate literacy, with spillover effects on the former function. In a large-scale developmental study, Santi et al. (2015) show that the impact of learning to read on visual skills is not observed at a macro behavioral level assessed with general educational/neuropsychological tests. Note, however, that studies that reported an impact of literacy on general spatial skills have examined children learning to read scripts differing on visual complexity (e.g., Zhou et al., 2014, in this research topic), but this was not the case in Santi et al.: all children were learning the alphabetic English orthography. This might seem, however, inconsistent with the *neuronal recycling hypothesis* (Dehaene, 2009). Indeed, a key question, discussed in the last four articles of this collection, is to understand which aspects of visual processing are actually affected by literacy acquisition and why. Possibly only the visual properties that collide with learning to read are affected. This is the case of *mirror invariance*: lateral mirror images, such as d and b, are originally processed as equivalent percepts. Kolinsky and Fernandes (2014; following the prior work of Pegado et al., 2014) examined whether learning to read is able to modify the object recognition system as expressed by a loss of mirror invariance, by comparing the orientation cost for mirror images (e.g., ⌉-⌈) vs. plane-rotations (to which the visual system is originally sensitive to; e.g., ⌉-⌊), in identity-based same-different judgments of illiterate, late literate, and early literate adults. In the same vein, using transcranial magnetic stimulation (TMS) during identity-based same-different judgments, Nakamura et al. (2014) demonstrated the causal role of the left occipitotemporal cortex (comprising the VWFA) in mirror discrimination of visual words by literate Japanese adults. In their opinion article, Pegado et al. (2014) set a multisystem learning framework to answer how mirror discrimination is acquired during learning to read. They propose that a tight functional link between the visual and motor systems is crucial for this acquisition. Finally, given that literacy acquisition also impacts on face recognition due to competition for neural space (cf. Dehaene, 2009), in an opinion article, Ventura (2014) reviews these evidence, discusses the possible reasons for this competition, and proposes new directions considering literacy as a form of visual expertise.

Taken together, these articles represent an update overview and demonstrate the diversity of approaches in this research topic: miscellaneous scientific backgrounds (e.g., neuroscience, in Nakamura et al., 2014; neuropsychology, in Qian and Bi, 2014; developmental psychology, in Santi et al., 2015; experimental psychology; in Kolinsky and Fernandes, 2014), several techniques (e.g., TMS, Nakamura et al., 2014; behavioral tests, Lachmann et al., 2014; item response models, Santi et al., 2015), various written scripts considered (i.e., studies with alphabetic and non-alphabetic scripts; e.g., Lachmann et al., 2014; Nakamura et al., 2014, respectively), different populations examined (typical vs. dyslexic readers, in Qian and Bi, 2014; adults of varying schooling and literacy levels, in Kolinsky and Fernandes, 2014). These articles are Dejerine's legacy as pieces of the (still incomplete) puzzle on the impact of literacy on visual processing, which will hopefully contribute to understand the reasons behind this impact.

## Conflict of interest statement

The authors declare that the research was conducted in the absence of any commercial or financial relationships that could be construed as a potential conflict of interest.
